# Supplementary material for: Examining linguistic shifts between preprints and publications
Source: PLoS Biol. 2022 Feb 1;20(2):e3001470. doi: 10.1371/journal.pbio.3001470 (PMC8806061; doi:10.1371/journal.pbio.3001470)
Supplement: S1 Text — (DOCX) [file pbio.3001470.s001.docx]

Document embeddings provide a means to categorize the language of documents in a way that takes into account the similarities between terms [[37](#ref-SdcIDSwR),[69](#ref-17IHBAq05),[70](#ref-14k28UnQN)]. We found that the first two PCs separated articles from different author-selected categories ([S1](#bookmark=id.1fob9te) Fig A). Certain neuroscience papers appeared to be more associated with the cellular biology direction of PC1, while others seemed to be more associated with the informatics-related direction ([S1](#bookmark=id.1fob9te) Fig A). This suggests that the concepts captured by PCs were not exclusively related to their field.

Visualizing token-PC similarity revealed tokens associated with certain research approaches ([S1](#bookmark=id.1fob9te) Fig B and [S1](#bookmark=id.1fob9te) Fig C). Token association of PC1 shows the separation of cell biology and informatics-related fields through tokens: “empirical”, “estimates” and “statistics” depicted in orange and “cultured” and “overexpressing” shown in blue ([S1](#bookmark=id.1fob9te) Fig B and [S2](#bookmark=id.2et92p0) Table). Association for PC2 shows the separation of bioinformatics and neuroscience via tokens: “genomic”, “genome” and “genomes” depicted in orange and “evoked”, “stimulus” and “stimulation” shown in blue ([S1](#bookmark=id.1fob9te) Fig C and S3 Table).

Examining the value for PC1 across all author-selected categories revealed an ordering of fields from cell biology to informatics-related disciplines ([S1](#bookmark=id.1fob9te) Fig D). These results suggest that a primary driver of the variability within the language used in bioRxiv could be the divide between informatics and cell biology approaches. A similar analysis for PC2 suggested that neuroscience and bioinformatics present a similar language continuum ([S1](#bookmark=id.1fob9te) Fig E). This result supports the notion that bioRxiv contains an influx of neuroscience and bioinformatics-related research results. For both of the top two PCs, the submitter-selected category of systems biology preprints was near the middle of the distribution and had a relatively large interquartile range when compared with other categories ([S1](#bookmark=id.1fob9te) Fig D and [S1](#bookmark=id.1fob9te) Fig E), suggesting that systems biology is a broader subfield containing both informatics and cellular biology approaches.

Examining the top five highest-scoring and bottom five lowest-scoring systems biology preprints along PC1 reinforces its dichotomous theme (S1 Table). Preprints with the highest values [[71](#bookmark=id.44sinio),[72](#bookmark=id.2jxsxqh),[73](#bookmark=id.z337ya),[74](#bookmark=id.3j2qqm3),[75](#bookmark=id.1y810tw)] included software packages, machine learning analyses, and other computational biology manuscripts, while preprints with the lowest values [[76](#bookmark=id.4i7ojhp),[77](#bookmark=id.2xcytpi),[78](#bookmark=id.1ci93xb),[79](#bookmark=id.3whwml4),[80](#bookmark=id.2bn6wsx)] were focused on cellular signaling and protein activity. We provide the rest of our 50 generated PCs in our online repository (see Software and Data Availability).

69. Chen M. Efficient Vector Representation for Documents through Corruption [Internet]. arXiv. arXiv; 2017 Jul. Report No.: 1707.02377. Available from: <https://arxiv.org/abs/1707.02377>

70. Gourru A, Guille A, Velcin J, Jacques J. Document Network Projection in Pretrained Word Embedding Space [Internet]. arXiv. arXiv; 2020 Jan. Report No.: 2001.05727. Available from: <https://arxiv.org/abs/2001.05727>

71. Bianconi F, Antonini C, Tomassoni L, Valigi P. Conditional Robust Calibration (CRC): a new computational Bayesian methodology for model parameters estimation and identifiability analysis. Cold Spring Harbor Laboratory [Internet]. 2017 Oct 2; Available from: <https://doi.org/gg9393>

72. Santos G, Vera J. <i>FPtool</i> a software tool to obtain <i>in silico</i> genotype-phenotype signatures and fingerprints based on massive model simulations. Cold Spring Harbor Laboratory [Internet]. 2018 Feb 18; Available from: <https://doi.org/gjr9m9>

73. Tankhilevich E, Ish-Horowicz J, Hameed T, Roesch E, Kleijn I, Stumpf MP, et al. GpABC: a Julia package for approximate Bayesian computation with Gaussian process emulation. Cold Spring Harbor Laboratory [Internet]. 2019 Sep 18; Available from: <https://doi.org/gg94bj>

74. Henkel R, Hoehndorf R, Kacprowski T, Knüpfer C, Liebermeister W, Waltemath D. Notions of similarity for computational biology models. Cold Spring Harbor Laboratory [Internet]. 2016 Mar 21; Available from: <https://doi.org/gg939z>

75. Dalle Pezze P, Le Novère N. SBpipe: a collection of pipelines for automating repetitive simulation and analysis tasks. Cold Spring Harbor Laboratory [Internet]. 2017 Feb 9; Available from: <https://doi.org/gg9392>

76. Kozuka C, Sales V, Osataphan S, Yuchi Y, Chimene-Weiss J, Mulla C, et al. Bromodomain inhibition reveals FGF15/19 as a target of epigenetic regulation and metabolic control. Cold Spring Harbor Laboratory [Internet]. 2019 Dec 12; Available from: <https://doi.org/gjr9m8>

77. Purvis GSD, Collino M, Tavio HMA, Chiazza F, O’Riodan CE, Zeboudj L, et al. Inhibition of Bruton’s tyrosine kinase reduces NF-kB and NLRP3 inflammasome activity preventing insulin resistance and microvascular disease. Cold Spring Harbor Laboratory [Internet]. 2019 Aug 28; Available from: <https://doi.org/gg94bg>

78. Selkrig J, Li N, Bobonis J, Hausmann A, Sueki A, Imamura H, et al. Spatiotemporal proteomics uncovers cathepsin-dependent host cell death during bacterial infection. Cold Spring Harbor Laboratory [Internet]. 2018 Nov 7; Available from: <https://doi.org/gg94bc>

79. Joly JH, Delfarah A, Phung PS, Parrish S, Graham NA. NADPH consumption by L-cystine reduction creates a metabolic vulnerability upon glucose deprivation. Cold Spring Harbor Laboratory [Internet]. 2019 Aug 13; Available from: <https://doi.org/gg94bf>

80. Zheng D, Sussman JH, Jeon MP, Parrish ST, Delfarah A, Graham NA. AKT but not MYC promotes reactive oxygen species-mediated cell death in oxidative culture. Cold Spring Harbor Laboratory [Internet]. 2019 Sep 1; Available from: <https://doi.org/gg94bh>
