# Supplementary material for: Examining linguistic shifts between preprints and publications
Source: PLoS Biol. 2022 Feb 1;20(2):e3001470. doi: 10.1371/journal.pbio.3001470 (PMC8806061; doi:10.1371/journal.pbio.3001470)
Supplement: S1 Table — (DOCX) [file pbio.3001470.s003.docx]

| Title [citation] | PC1 | License | Figure Thumbnail |
| --- | --- | --- | --- |
| Conditional Robust Calibration (CRC): a new computational Bayesian methodology for model parameters estimation and identifiability analysis [[71](#bookmark=id.44sinio)] | 4.522818390064091 | None | 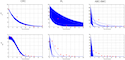 |
| FPtool a software tool to obtain in silico genotype-phenotype signatures and fingerprints based on massive model simulations [[72](#ref-Maazaz8h)] | 4.348956760251298 | CC-BY | 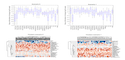 |
| GpABC: a Julia package for approximate Bayesian computation with Gaussian process emulation [[73](#ref-16tWubqOj)] | 4.259104249060651 | CC-BY-NC-ND | 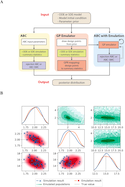 |
| Notions of similarity for computational biology models [[74](#ref-UmFNlo8W)] | 4.079855550647664 | CC-BY-NC-ND | 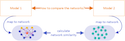 |
| SBpipe: a collection of pipelines for automating repetitive simulation and analysis tasks [[75](#ref-6gfAR2hM)] | 4.022240241143516 | CC-BY-NC-ND | 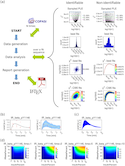 |
|  |  |  |  |
| Bromodomain inhibition reveals FGF15/19 as a target of epigenetic regulation and metabolic control [[76](#ref-NIw2ZKUa)] | -3.4783803547922414 | None | 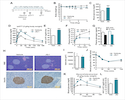 |
| Inhibition of Bruton’s tyrosine kinase reduces NF-kB and NLRP3 inflammasome activity preventing insulin resistance and microvascular disease [[77](#ref-1CM9H3mFb)] | -3.6926161167521476 | None | 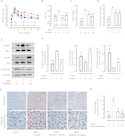 |
| Spatiotemporal proteomics uncovers cathepsin-dependent host cell death during bacterial infection [[78](#ref-2V2BJL87)] | -3.728443135960558 | CC-BY-ND | 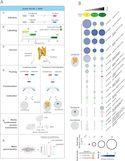 |
| NADPH consumption by L-cystine reduction creates a metabolic vulnerability upon glucose deprivation [[79](#ref-nzDb3Lvn)] | -3.7363965062637288 | None | 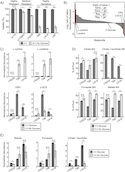 |
| AKT but not MYC promotes reactive oxygen species-mediated cell death in oxidative culture [[80](#ref-jmFU2eta)] | -3.8769231933681176 | None | 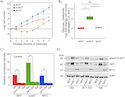 |

71. Bianconi F, Antonini C, Tomassoni L, Valigi P. Conditional Robust Calibration (CRC): a new computational Bayesian methodology for model parameters estimation and identifiability analysis. Cold Spring Harbor Laboratory [Internet]. 2017 Oct 2; Available from: <https://doi.org/gg9393>

72. Santos G, Vera J. <i>FPtool</i> a software tool to obtain <i>in silico</i> genotype-phenotype signatures and fingerprints based on massive model simulations. Cold Spring Harbor Laboratory [Internet]. 2018 Feb 18; Available from: <https://doi.org/gjr9m9>

73. Tankhilevich E, Ish-Horowicz J, Hameed T, Roesch E, Kleijn I, Stumpf MP, et al. GpABC: a Julia package for approximate Bayesian computation with Gaussian process emulation. Cold Spring Harbor Laboratory [Internet]. 2019 Sep 18; Available from: <https://doi.org/gg94bj>

74. Henkel R, Hoehndorf R, Kacprowski T, Knüpfer C, Liebermeister W, Waltemath D. Notions of similarity for computational biology models. Cold Spring Harbor Laboratory [Internet]. 2016 Mar 21; Available from: <https://doi.org/gg939z>

75. Dalle Pezze P, Le Novère N. SBpipe: a collection of pipelines for automating repetitive simulation and analysis tasks. Cold Spring Harbor Laboratory [Internet]. 2017 Feb 9; Available from: <https://doi.org/gg9392>

76. Kozuka C, Sales V, Osataphan S, Yuchi Y, Chimene-Weiss J, Mulla C, et al. Bromodomain inhibition reveals FGF15/19 as a target of epigenetic regulation and metabolic control. Cold Spring Harbor Laboratory [Internet]. 2019 Dec 12; Available from: <https://doi.org/gjr9m8>

77. Purvis GSD, Collino M, Tavio HMA, Chiazza F, O’Riodan CE, Zeboudj L, et al. Inhibition of Bruton’s tyrosine kinase reduces NF-kB and NLRP3 inflammasome activity preventing insulin resistance and microvascular disease. Cold Spring Harbor Laboratory [Internet]. 2019 Aug 28; Available from: <https://doi.org/gg94bg>

78. Selkrig J, Li N, Bobonis J, Hausmann A, Sueki A, Imamura H, et al. Spatiotemporal proteomics uncovers cathepsin-dependent host cell death during bacterial infection. Cold Spring Harbor Laboratory [Internet]. 2018 Nov 7; Available from: <https://doi.org/gg94bc>

79. Joly JH, Delfarah A, Phung PS, Parrish S, Graham NA. NADPH consumption by L-cystine reduction creates a metabolic vulnerability upon glucose deprivation. Cold Spring Harbor Laboratory [Internet]. 2019 Aug 13; Available from: <https://doi.org/gg94bf>

80. Zheng D, Sussman JH, Jeon MP, Parrish ST, Delfarah A, Graham NA. AKT but not MYC promotes reactive oxygen species-mediated cell death in oxidative culture. Cold Spring Harbor Laboratory [Internet]. 2019 Sep 1; Available from: <https://doi.org/gg94bh>
