# Supplementary figures and images for: Examining linguistic shifts between preprints and publications
Source: PLoS Biol. 2022 Feb 1;20(2):e3001470. doi: 10.1371/journal.pbio.3001470 (PMC8806061; doi:10.1371/journal.pbio.3001470)

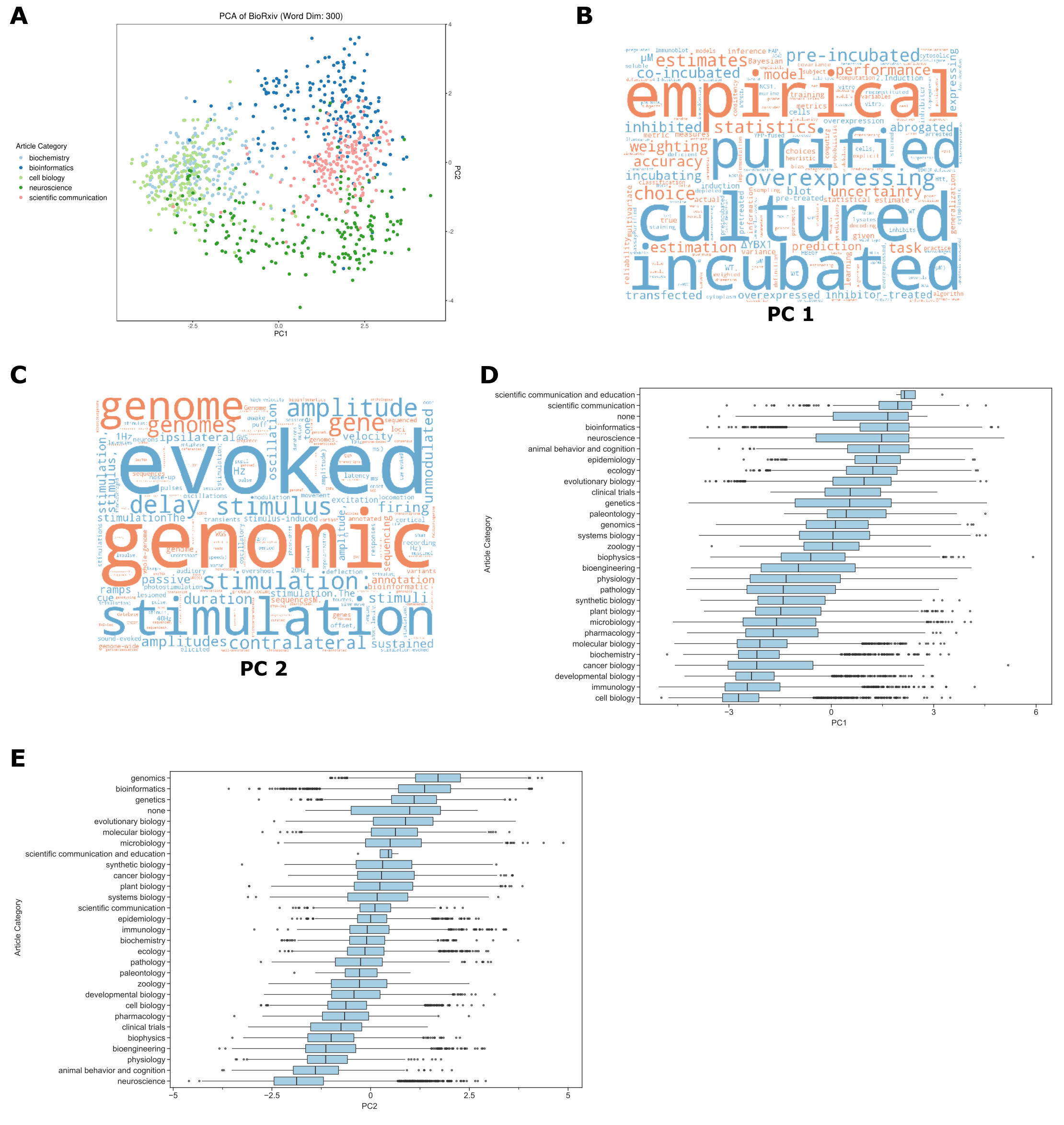

Supplement: S1 Fig — (A) PCA of bioRxiv word2vec embeddings groups documents based on author-selected categories. We visualized documents from key categories on a scatterplot for the first 2 PCs. The first PC separated cell biology from informatics-related fields, and the second PC separated bioinformatics from neuroscience fields. (B) A word cloud visualization of PC1. Each word cloud depicts the cosine similarity score between tokens and the first PC. Tokens in orange were most similar to the PC’s positive direction, while tokens in blue were most similar to the PC’s negative direction. The size of each token indicates the magnitude of the similarity. (C) A word cloud visualization of PC2, which separated bioinformatics from neuroscience. Similar to the first PC, tokens in orange were most similar to the PC’s positive direction, while tokens in blue were most similar to the PC’s negative direction. The size of each token indicates the magnitude of the similarity. (D) Examining PC1 values for each article by category created a continuum from informatics-related fields on the top through cell biology on the bottom. Specific article categories (neuroscience and genetics) were spread throughout PC1 values. (E) Examining PC2 values for each article by category revealed fields like genomics, bioinformatics, and genetics on the top and neuroscience and behavior on the bottom. PC, principal component; PCA, principal component analysis. (TIFF) [file pbio.3001470.s007.tiff]

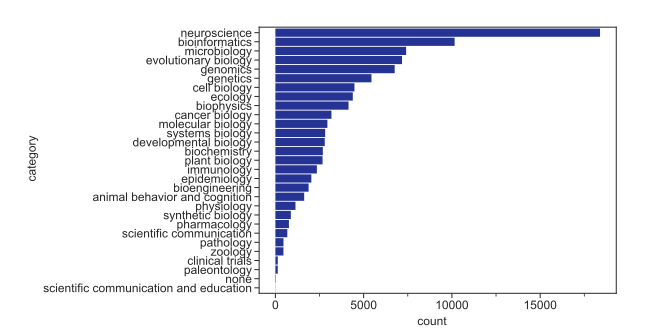

Supplement: S2 Fig — (TIFF) [file pbio.3001470.s008.tiff]

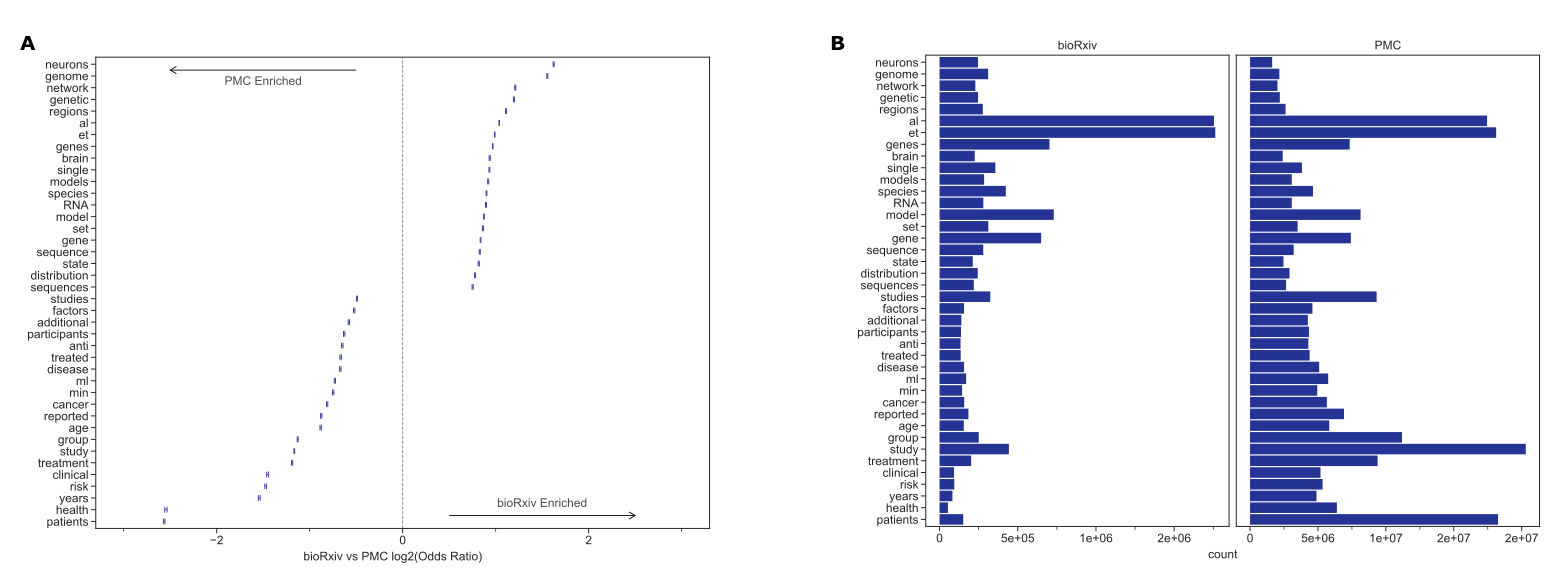

Supplement: S3 Fig — (A) The significant differences in token frequencies for the corpora appear to be driven by the fields with the highest uptake of bioRxiv, as terms from neuroscience and genomics are relatively more abundant in bioRxiv. We plotted the 95% confidence interval for each reported token. (B) Of the tokens that differ between bioRxiv and PMC, the most abundant in bioRxiv are “gene,” “genes,” and “model,” while the most abundant in PMC is “study.” PMC, PubMed Central. (TIFF) [file pbio.3001470.s009.tiff]

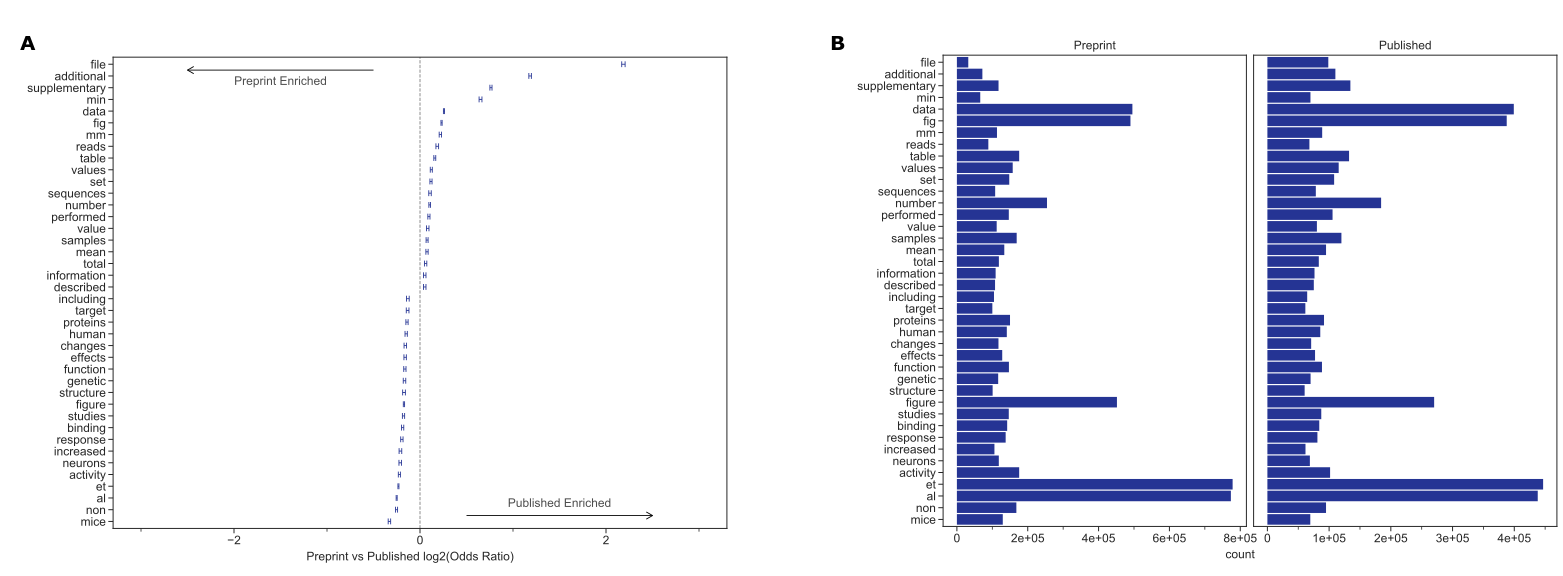

Supplement: S4 Fig — (A) The significant differences in token frequencies for preprints and their corresponding published version often appear to be associated with data availability and supporting information or additional materials. We plotted the 95% confidence interval for each reported token. (B) The tokens with the largest absolute differences in abundance appear related to scientific figures and data availability. (TIFF) [file pbio.3001470.s010.tiff]

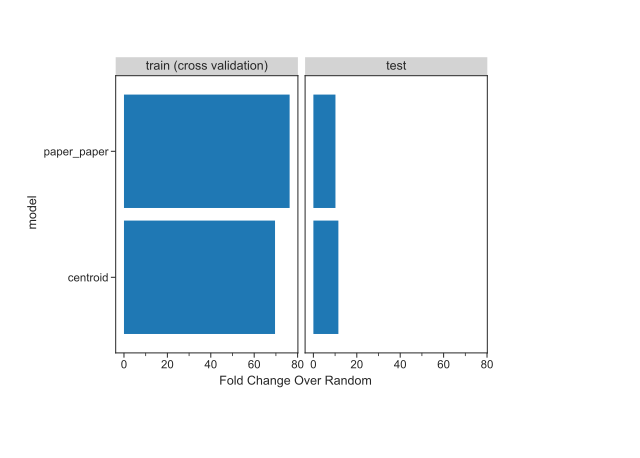

Supplement: S5 Fig — This bargraph shows each model’s accuracy in respect to predicting the training and test set. (TIFF) [file pbio.3001470.s011.tiff]
