# Supplementary material for: Examining linguistic shifts between preprints and publications
Source: PLoS Biol. 2022 Feb 1;20(2):e3001470. doi: 10.1371/journal.pbio.3001470 (PMC8806061; doi:10.1371/journal.pbio.3001470)
Supplement: Attachment — Submitted filename: r2r.pdf [file pbio.3001470.s013.pdf]

Response to Reviewers

Reviewer #1

[identifies himself as Ross Mounce]

This manuscript ‘Linguistic Analysis of the bioRxiv Preprint Landscape’ presents an analysis of bioRxiv fulltexts and metadata, relative to journal-published versions of the same preprints (n= 17,952 pairs), and the New York Times Annotated Corpus.

It’s an interesting manuscript worthy of publication in PLOS Biology after a few relatively minor revisions.

I have left many comments directly on the manuscript via the dedicated manuscript website, using public Hypothes.is annotations:
https://greenelab.github.io/annorxiver_manuscript/

I incorporate some but not all of these comments into this formal review supplied to the journal (PLOS Biology) who invited me to review this manuscript.

Unsurprisingly, biorxiv preprints and journal-published versions of biorxiv preprints are found to be linguistically different to the New York Times Annotated Corpus e.g. in average document length and to a lesser degree in average sentence length, and % in passive voice.

Luckily there are plenty more actually interesting results reported in this manuscript, not least that of 23,271 preprint-published pairs, 17,952 of those pairs (>77%) had a published version present within the PMCOA corpus. I don’t think the authors quite realise the significance of this result. 77% is a very very high rate of open access. It could do with being discussed more within the manuscript e.g. relative to the overall (lower) rate of open access of *all* biomedical and life science research articles. What does this signify about preprint authors / ‘preprinters’?

I can think of a couple of hypotheses:

a) preprinters are perhaps more likely to have grant-funded research subject to an open access policy

b) perhaps preprinters are more publishing ‘savvy’ and want to achieve more impact/citations and thus strive harder to ensure that the eventual journal-published version of their work is open access (reflected in being in PMCOA).

We appreciate the reviewer’s positive comments on our manuscript. The reviewer is corrected that we did not realize that the finding of 77% of preprint-published pairs being present in PMCOA is a surprising discovery, and we thank this reviewer for bringing this

to our attention. We now include the following in our discussion section to emphasize this point:

```
+ Over 77% of bioRxiv preprints with a corresponding publication in ou
r snapshot were successfully detected within Pubmed Central's Open Acc
ess Corpus (PMCOA).
+ This suggests that most work from groups participating in the prepri
nt ecosystem is now available in final form for literature mining and
other applications.
```

> If it were my choice I would cut the entire subsection 'Document embeddings derived from bioRxiv reveal fields and subfields'. It is already known that document embeddings can reveal fields and subfields. Being 'preprints' or 'biorxiv preprints' rather than say published journal articles won't change that. I found this section very uninteresting and extremely un-novel. It is descriptive and accurate, but in the context of an already long manuscript, I feel it is unnecessary.

We felt that this analysis also included other findings that were less obvious: namely the principal components that separated fields and the finding that certain fields like systems biology were spread across certain components that distinguished quantitative systems biology from cellular systems biology papers. We think that this lays the groundwork for a number of future research efforts. However, we agree with the reviewer that this manuscript does present a broad examination of the full text content of *bioRxiv* and is somewhat lengthy, so we did move this section into the supplement.

> Aside from the manuscript, I have some brief comments on the actual web application.
>
> I tried some palaeontology preprints (it's a field i'm very familiar with). The results were rather mixed. e.g. for https://greenelab.github.io/preprint-similarity-search/?doi=10.1101/2020.12.10.406678 ("The first dinosaur egg remains a mystery"), the most similar paper recommendations were excellent. However, the most similar journals suggested were surprisingly poor - many of these could obviously at a glance never publish this preprint (dinosaurs are not plants!) e.g. American Journal of Botany, World Archaeology, Journal of Phycology, The Holocene, Botanical Journal of the Linnean Society. Linnean Society of London
>
> But I realise that PMCOA isn't exactly great training date for interpreting palaeontology articles – fringe content from PMC's perspective(?)

We agree that using PMCOA as a training set will limit the fields to which the website can be applied. We felt that PMCOA was likely to be appropriate for much *bioRxiv* and *medRxiv* content, which are the servers our tool supports. We have also implemented a system to automatically update to bring in new PMCOA papers. If PMCOA begins to include more journals that publish articles in these fields or author-contributed manuscripts in these fields, then the tool would be more likely to identify appropriate matches.

Specific comments:

1.) https://hypothes.is/a/1ODs5NLbEeuhnEPjCpUFpA

I think this needs to be made more specific as [25] analysed a few different things.

Your statement here is true with respect to their analysis of abstract text “Over 50% of abstracts had changes that minorly altered, strengthened, or softened the main conclusions”

BUT

it is not true with respect to the panels and tables analysis in [25]:

“over 70% of 162 published preprints were classified with “no change” or superficial rearrangements to panels and 163 tables, confirming the previous conclusion"

thus perhaps you should consider writing something like:

an analysis of preprints posted at the beginning of 2020 revealed that over 50% underwent minor changes in the abstract text as they were published, but over 70% had ‘no change’ or only superficial rearrangements to panels and tables [25].

We agree with the reviewer that the proposed phrasing is better. We now write:

```
- Preprint repositories by definition do not perform in-depth peer review, which can result in posted preprints containing inconsistent results or conclusions [...]; however, an analysis of preprints posted at the beginning of 2020 revealed that most underwent minor changes as they were published [...].

+ Preprint repositories by definition do not perform in-depth peer review, which can result in posted preprints containing inconsistent results or conclusions [...]; however, an analysis of preprints posted at the beginning of 2020 revealed that over 50% underwent minor changes in the abstract text as they were published, but over 70% did not change or only had simple rearrangements to panels and tables [...].
```

2.) https://hypothes.is/a/djNirNLcEeu2YxNM5WBxvA

but to clarify, you did remove these from the analysis, right? It would just be good to clarify that. They are easy to identify and should just be removed. I can’t see how they would add anything but noise to this analysis. What is the total number of preprints after withdrawn preprints are removed from the sample?

In the version of the manuscript that the reviewer saw, we did not remove these preprints from our analysis as we felt their impact would be minimal. Based on the reviewer’s comments, we have now rerun all of the analyses with these withdrawn articles removed.

This did not lead to substantive changes in the article or figures, but we agree it was the most rigorous analysis and are happy that the reviewer brought up this point.

```
- As there were very few withdrawn preprints, we did not treat these a
s a special case.

+ We encountered a total of 72 withdrawn preprints within our snapshot
.
+ After removal, we were left with 97,951 preprints for our downstream
analyses.
```

3.) https://hypothes.is/a/bmdkpNLeEeuZ0k8oLqyeWQ

actually, there need not necessarily be an embargo period. Many publishers now offer a zero-day embargo so that the author accepted manuscript can be deposited either at acceptance (before even journal publication!) or on the day of journal publication. Even if the journal normally tries to embargo the work, you can see some full text author manuscripts become immediately available well before the journal would normally permit them 'out' thanks to the Plan S Rights Retention Strategy e.g. this one here: https://www.ncbi.nlm.nih.gov/pmc/articles/PMC7610590/

So what you should really say here is that full text works appear in PMC as either accepted author manuscripts (green open access) or via open access publishing at the journal (gold open access).

BTW, I resent calling it a 'closed access' [article?] if the accepted manuscript is fully freely available – that would seem to give undue primacy to the journal published version. It's an article with different versions - one freely accessible at a repository e.g. PMC, without publisher branding and another behind a paywall at the publisher website with publisher branding

We agree that 'closed access' was imprecise phrasing. We have updated this section in our manuscript.

```
- PMC articles can be closed access ones from research funded by the N
IH appearing after an embargo period or be published under Gold Open A
ccess [...] publishing schemes.

+ Articles appear in PMC as either accepted author manuscripts (Green
Open Access) or via open access publishing at the journal (Gold Open A
ccess [...]).
```

4.) https://hypothes.is/a/-v_PbtLeEeu8Rw8afgzT5g

presuming a journal allows individual articles to be published with a CC BY licence under a so-called 'hybrid-OA' option, can a journal really NOT participate for those CC BY licenced articles? If biomedically relevant and CC BY licensed surely PMC takes that

content at the article level and thus its debatable as to whether journals really have the power to actually 100% not participate.

We agree with the reviewer that this is an important distinction and have adjusted our text:

```
- Individual journals have the option to fully participate in submitti
ng articles to PMC, selectively participate sending only a few papers
to PMC, only submit papers according to NIH's public access policy [..
.], or not participate at all.

+ Individual journals have the option to fully participate in submitti
ng articles to PMC, selectively participate sending only a few papers
to PMC, only submit papers according to NIH's public access policy [..
.], or not participate at all; however, individual articles published
with the CC BY license may be incorporated.
```

5.) https://hypothes.is/a/vekhYNLfEeuxGm9i6MkLqw it's a real pity you chose not to compare preprints to author manuscripts. As your results demonstrated, lots of the word changes were just journal-style related e.g. "figure" -> "fig." . An analysis of just preprints matched to author manuscripts would get more closely and cleanly to what the textual difference between pre-peer-review and post-peer-review (without minor stylistic changes).

We agree that contrasting differences in bioRxiv to author-supplied vs journal-supplied manuscripts have the potential to distinguish journal-related stylistic from author-related ones. We felt that this would be an interesting manuscript in its own right and that we would not be able to give it an appropriate treatment within this manuscript.

6.) https://hypothes.is/a/YkqPKNLgEeucPGc1_W-jzA

minor typo: tagging surely

Thank you for pointing this out. We updated text to fix this typo.

```
- This collection contains over 1.8 million articles where 1.5 million
of those articles have undergone manual entity tagged by library scien
tists [...].

+ This collection contains over 1.8 million articles where 1.5 million
of those articles have undergone manual entity tagging by library scie
ntists [...].
```

7.) https://hypothes.is/a/Bsv68tLkEeugHEfCZMUGag From the perspective of a person (me!) interested in open access to ('final') peer-reviewed research outputs this is a super interesting result in itself, which should perhaps be remarked upon more in this manuscript.

It implies that over 77% (17,952/23,271) of biomedical preprints that are detectably linked to a journal published paper, that subsequent journal published paper became open access in the PMCOA corpus (regardless of specific means/route). That's great news. The subset of works from biomedical researchers that do preprinting have a much higher level of open access (to the eventual journal published version) than biomedical research overall (including works that don't have a preprint version)

See figure 3a from 'Open access levels: a quantitative exploration using Web of Science and oaDOI data' by Bosman and Kramer for a comparator looking at OA levels in biomedical and life science papers https://peerj.com/preprints/3520.pdf even in the 'best' OA performing subfield (Cell Biology) it doesnt reach 70%. 30% to 50% is more typical albeit looking at 2016 publications.

Put another way, we only 'lose' 23% of biomedical preprinted research to paywalled journals that do not allow a copy of the work to be made full text available in the PMCOA corpus, in reasonable time**. And in those cases we still have access to the preprint

** with no doubt many other caveats such as cases where the author could do it without help from the journal, but does not for some unknown reason

We appreciate that the reviewer pointed out this interesting finding in our results that we had missed, and we have updated our discussion accordingly.

```
- The majority of research involving bioRxiv focuses on the metadata o
f preprints; however, the language contained within these preprints ha
s not previously been systematically examined.

+ Over 77% of bioRxiv preprints with a corresponding publication in ou
r snapshot were successfully detected within Pubmed Central's Open Acc
ess Corpus (PMCOA).
+ This suggests that most work from groups participating in the prepri
nt ecosystem is now available in final form for literature mining and
other applications.
+ Most research on bioRxiv preprints has examined their metadata; we e
xamine the text content as well.
```

8.) https://hypothes.is/a/aWmt3tLIEeuh3M-Ku8ThvQ

To be clear 326 stopwords is the default setting?

Interestingly 'ca' is one of those 326 stopwords. I would have thought that one might actually be significant in a life sciences context e.g. calcium channels "Ca2+"

We used the 326 stopwords provided by default. We agree that the stopwords are not precisely tuned for life sciences research. We have adjusted our text to say:

```
- We used spaCy's "en_core_web_sm" model [@spacy2] (version 2.2.3) to
preprocess all corpora and filter out 326 spaCy-provided stopwords.
```

```
+ We used spaCy's "en_core_web_sm" model [@spacy2] (version 2.2.3) to preprocess all corpora and filter out 326 stopwords using spaCy's default settings.
```

9.) https://hypothes.is/a/dkIurtLmEeuqkU-qTN4dtg

I'm sure you've got this in the github, but just to make the manuscript more readily understandable without digging around in github, do you think you could provide as a supplementary file a list of those 100 most frequently occurring tokens, so that people can get a better feel for what the data is here?

We agree with the reviewer that this is a convenient table to have at hand. This is now Supplementary Table 5.

10.) https://hypothes.is/a/-2AU8tLmEeu6swvY4jN5Mw

hmmm… not a problem of this manuscript, but that's really not good enough from bioRxiv is it? Change one word of a long and complex title and suddenly 'oh, we can't do it'. A comment to suggest that bioRxiv could do better would be fun, no(?) i.e. look at author names AND title and if both are similar enough, then do auto-linking.

oh okay, you did actually do that. Nice :)

We are glad that you liked our document matching solution.

11.) https://hypothes.is/a/dhGbgNLnEeuYkkfUNqKwgA

I don't suppose you could possibly be precise about this rather than just 'a limited number'? Is it 5, 50, or 500?

We agree that this line is vague and have updated our text to be more explicit.

```
- There were a limited number of cases in which authors appeared to post preprints after the publication date, which results in preprints receiving a negative time difference, as previously reported [...]

+ We encountered 123 cases where the preprint posting date was subsequent to the publication date, resulting in a negative time difference, as previously reported [...].
```

12.) https://hypothes.is/a/DzrzGtLtEeuJlcdOlUfUBA

I'm surprised to see no citation given to JANE: https://jane.biosemantics.org/

https://academic.oup.com/bioinformatics/article/24/5/727/202224

reviewed in https://www.ncbi.nlm.nih.gov/pmc/articles/PMC6300233/

The 'find journals' functionality of JANE appears somewhat similar to the discovering similar journals functionality here.

Thank you for pointing this citation out. We have incorporated it into our manuscript.

```
- We developed a web application that places any bioRxiv or medRxiv pr
eprint into the overall document landscape and identifies similar pape
rs and journals.

+ We developed a web application that places any bioRxiv or medRxiv pr
eprint into the overall document landscape and identifies topically si
milar papers and journals (similar to [..]).
```

13.) https://hypothes.is/a/eJ7-DtLpEeuh0A8OL8WAWw

This is the kind of journal-faff difference that I hypothesise would not be visible or less visible if one did an analysis of preprints vs author manuscripts.

There is change but is change from figure to 'fig.' to suit journal style actually helpful/valuable? In my opinion it is not!

We recognize the limitation of our design choice and agree it would be interesting to compare journal-supplied vs author-supplied manuscripts in a future study.

14.) https://hypothes.is/a/-ixA_NLqEeuv6YviR-Il0A

I would cut this entire subsection from the manuscript to make it shorter (or relegate it to a supplementary section).

Don't we already know that if one uses full texts we can determine the subfield of the paper? It's not that interesting in my opinion and not relevant to the main hypotheses of the paper – comparing between preprints and the journal published version.

We believe this section is needed to describe the landscape of the bioRxiv corpus; however, we agree the manuscript is long and has many results, so we moved this section to the supplement.

15.) https://hypothes.is/a/V2wj3NLqEeuZ9idQ44yS4Q

adjusting for recency? i.e. not sampling 2019 preprints? in figure C the line indicates (if I'm interpreting correctly) that overall only 46.55% are published but that's because it includes very recent preprints that haven't had time to be journal published yet. Just be explicit that you are adjusting for recency (i.e. excluding 2019 and newer preprints) when you say that most preprints are eventually published.

Initially, we incorporated all preprints (2013 to early 2020) when calculating the overall proportion of preprints published. We agree that adjusting for recency is a better approach; however, we need to incorporate all preprints to have a fair comparison

against Abdill et al. estimate. The authors' estimate incorporated all preprints at the time of their study. Therefore, we updated our analysis to include two proportion estimates. Our first estimate included all preprints posted onto bioRxiv, while the second estimate only considers preprints posted before 2019. These two estimates are found in Figure 2 in our updated manuscript (reproduced below), where the first estimate is the solid green line and our second estimate is the dashed line.

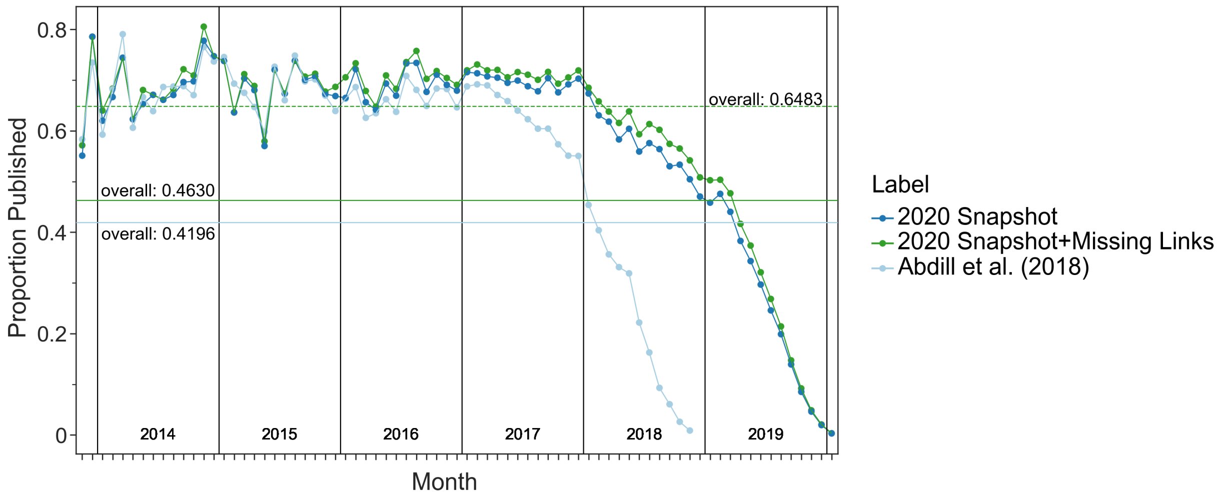

updated overall proportion calculation

16.) https://hypothes.is/a/N8cKltLrEeuZ-iPc5lrP5g

text changes relative to the journal published version? You might want to make that more explicit. Text changes alone is not adequately specific in my opinion.

We agree that text changes were vague and have updated the title accordingly.

```
- Preprints with more versions or more text changes took longer to pub
lish

+ Preprints with more versions or more text changes relative to their
published counterpart took longer to publish
```

17.) https://hypothes.is/a/z4r5ztLgEeupmPfOPmlVbw think this is insufficient information.

It should be more clearly highlighted that the NYTAC is proprietary data and it may require a fee of $150-300 to be paid to access, if a non-member of the Linguistic Data Consortium. To say merely "is available upon request" and nothing else is not quite true to my eyes - please warn that it may require payment to access, depending on one's institutional affiliation (or lack thereof).

We agree and have updated this text to make the fees for accessing NYTAC more apparent.

```
- Access to the New York Times Annotated Corpus (NYTAC) is available u
pon request with the Linguistic Data Consortium at [https://catalog.ld
c.upenn.edu/LDC2008T19](https://catalog.ldc.upenn.edu/LDC2008T19).

+ New York Times Annotated Corpus (NYTAC) can be accessed from the Lin
guistic Data Consortium at [https://catalog.ldc.upenn.edu/LDC2008T19](
https://catalog.ldc.upenn.edu/LDC2008T19) where there may be a \$150 t
o \$300 fee depending on membership status.
```

> is using a proprietary data set that charges for access congruent with the PLOS data availability policy?
>
> See: “Please note, if data have been obtained from a third-party source, we require that other researchers would be able to access the data set in the same manner as the authors” https://journals.plos.org/plosone/s/data-availability despite that URL indicating just PLOS ONE, the policy applies to all PLOS journals, unless otherwise noted.

We believe that using the NYTAC dataset does not violate PLOS’s data availability policy. We accessed the dataset in accordance with this policy.

Reviewer #2

> Overall, I enjoyed this manuscript for offering a way to quantify the transition of preprints to manuscripts within the biological sciences. Further, the authors develop an approach that could also more generally be useful for classifying biomedical literature, and they even provide as an example a web-based program to find potential publication avenues.

We appreciate the reviewer’s positive sentiment about our work.

> The methodological approach of the authors is quite unexpected. While I do not see a fundamental flaw in their approach, I would anticipate it to be biased toward the most frequent phrases. When performing computational research there is a risk to pursue analyses through well-intended “improvements” or “customizations” whenever the approach does not seem to yield the expected outcome. As some people could be tempted to interpret parts of the analysis of the authors as warning flags for above having happened, I would recommend adding some additional control analyses and explicit statements about their chosen rationales.
>
> Particularly, I would be very curious about the discussion, or main text commenting on why the authors created a custom scheme of classifying documents and their similarity based on vectors of words instead of using existing approaches that provide vectors of documents - including doc2vec that is included in the software package that the authors used for word2vec. Do the results change according to the approach?

We agree with the reviewer that there are a number of possible approaches to both explore the linguistic landscape of bioRxiv and to predict the ultimate publication venue of preprints. Our first goal was to examine the linguistic landscape, so we prioritized

strategies that led to interpretable embedding spaces. Our primary strategy was to use word embeddings, which we could use to explore topics and language. We combined those same word embeddings to produce document embeddings as the average of the word embeddings. This lets us use the same underlying embeddings through the manuscript from the exploration of language through the journal prediction. We also agree with the reviewer that Doc2Vec would be likely to produce embeddings that would improve performance on the prediction task.

For this revision, we directly compared both methods for their interpretability and predictive performance. We find that the Word2Vec model's first principal component (shown below) was more interpretable than the one from the Doc2Vec model (also below). On the other hand, we find that the Doc2Vec model's predictive accuracy is better for the journal classification task (also below). On balance, we think including multiple embedding models within this paper, which aims to characterize the linguistic landscape of bioRxiv, would be confusing. For this revision, we have elected to retain the Word2Vec model. We debated changing the journal prediction server and results to use Doc2Vec, but also considering the feedback from reviewer #1 about the complexity of the paper decided against at this time. We could easily imagine an improvement to the server in the future that moves to Doc2Vec.

Word2Vec 1st Principal Component WordCloud

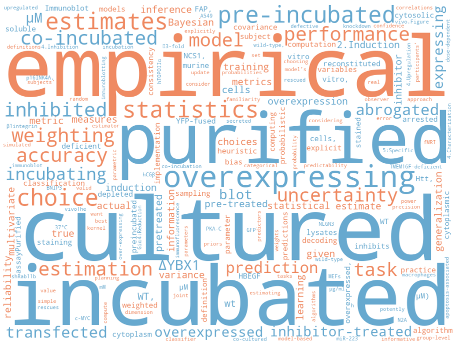

Doc2Vec 1st Principal Component WordCloud

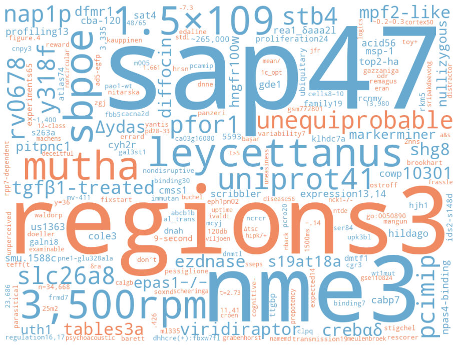

Doc2vec vs Word2vec in Journal Recommendations

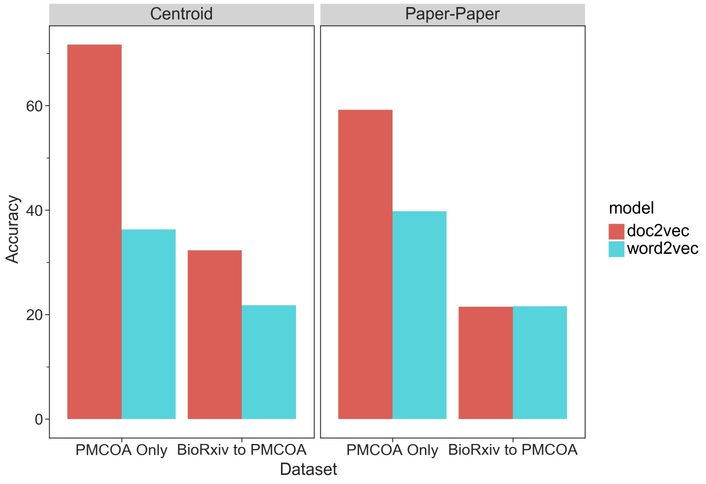

> Further, word2vec often seems to work even better when first trained on a larger corpus before then being applied or transferred to more specialized corpora. Personally, I also made this experience when following an example tutorial provided by the creators of the package that the authors used - which too suggests starting with existing pre-trained models. While the more restricted training done by the authors might have reduced the sensitivity of their approach (… which would likely only strengthen their claims), I would be curious whether there was an additional rationale for avoiding the former strategy that might be missed by readers (e.g.: different meanings such as “abstract” that has different meanings for scientists and non-scientists?).

We did consider using a pretraining step, but we were worried that then it might be hard to differentiate the effect of pretraining from the text content of bioRxiv. Since our goal was to explore the linguistic landscape of bioRxiv itself, we elected not to do this. We agree with the reviewer that if the goal is to enhance the predictive performance of the similarity search webserver, this would be a good step to take. As with above, we may consider this in a future iteration of the server, but at this point we want to make sure the work continues to characterize the linguistic landscape of bioRxiv.

> Likewise, I’m wondering why the authors used a Euclidean distance for word embeddings instead of a Cosine similarity (which if I recall correctly would also be default in the similarity module of the package which the authors used). Cosine similarity should also allow the authors to make statements about the similarity of words without imposing assumptions on similar text lengths or usage frequencies.

We used Euclidean distance because it satisfied the triangle inequality, which let us use the sklearn implementation of k-d tree for preprint similarity search. This allowed us to perform search efficiently with a minimum of development time, and also provided a framework that we could use to shard the search across nodes in the event that we needed to further accelerate performance. We didn’t find the distance metric to be a substantial driver of performance: preprints and their published counterparts have significantly lower distances regardless of the distance metric used (comparison figure provided below).

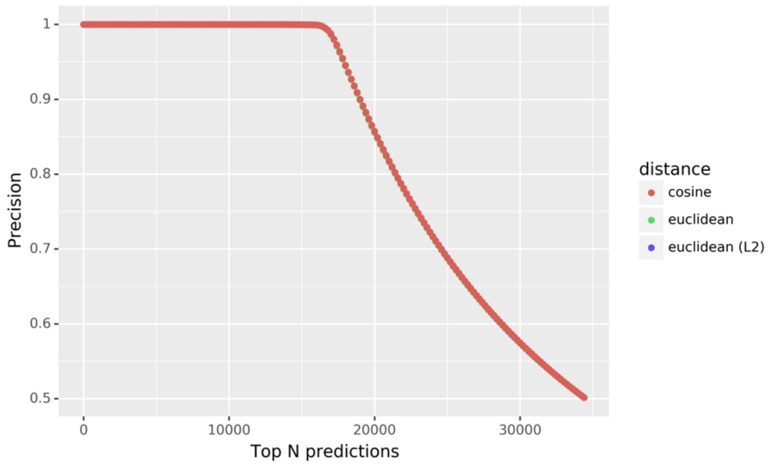

Distance comparison

> Similarly, I was wondering how the “journal-based” approach, which the authors mention briefly against the influence of high publication frequency journals, was implemented. Further, if it could have been avoided by avoiding the Euclidean space.

> The mapping of similarity seems to be based on individual pairs of text and as such it would seem vulnerable of shifting distributions (e.g.: if published articles were somewhat different from preprints, as implied in Figure 1A). I would suspect that the authors would be able to improve their performance even further by doing global matching between many pairs (… again see their adherence to a weaker approach as something that ultimately strengthens their findings). Again, a comment on the rationale of their chosen approach could convey additional non-evident considerations.

While a more optimal distance metric could make some difference in performance, it wouldn’t substantially change the issue of the background frequency whereby some mega journals have published orders of magnitude more manuscripts than others. We feel that the two approaches together (nearest publications, nearest centroids) provide a reasonable solution that can be effectively integrated into a web interface. The reviewer’s additional comment about implementing an explicit adjustment for the distributional shift is quite insightful and would likely be a path to improved performance. We could imagine using something like orthogonal Procrustes to align the preprint-publication vector spaces before performing search. We also recently added the capability to automatically update the search indices, which makes implementing this more challenging (we’d want to check the alignments each time), so we didn’t implement this at this time but we agree with the reviewer that this is an important path to explore for improved performance in the future.

> I love the web application!

We are happy you like the application.

> No statistics are given for the enrichments in Figure 1B-E.

We plot each token with a 95% confidence interval (CI). The underlying frequency counts are sufficiently large that the confidence intervals are difficult to discern on the plot. We have adjusted our plot style to make the confidence intervals more apparent. This doesn't help Fig 1B so much as the counts remain very large, but the confidence intervals are now more apparent in Fig 1D.

I would welcome a supplemental analysis, that removes single letters and special characters from the analysis of Figure 1B-E as they might change the baseline.

We have added this analysis to our supplement (Supplemental Figures S3 and S4). We reproduced these figures below.

BioRxiv vs Pubmed Central Supplemental Figure S3

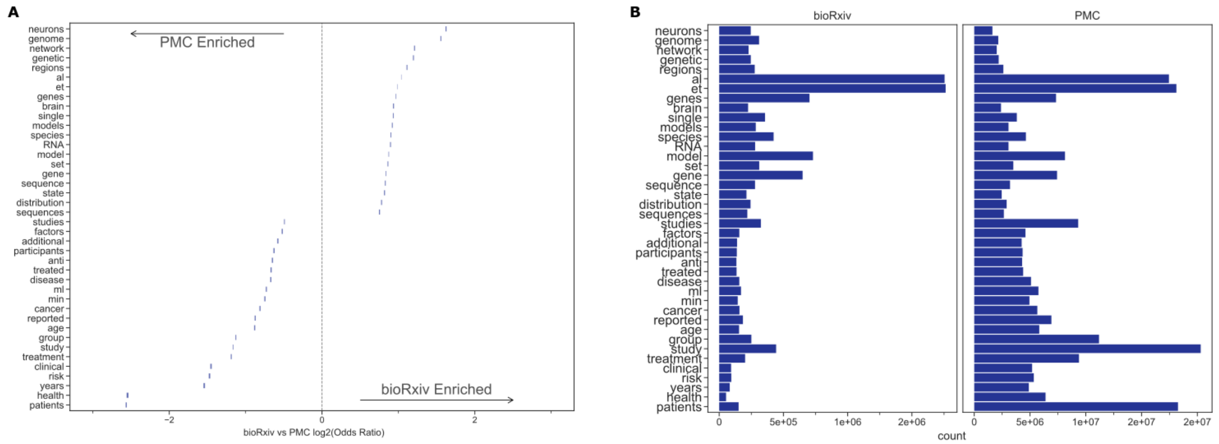

Preprints vs their Published Counterparts Supplemental Figure S4

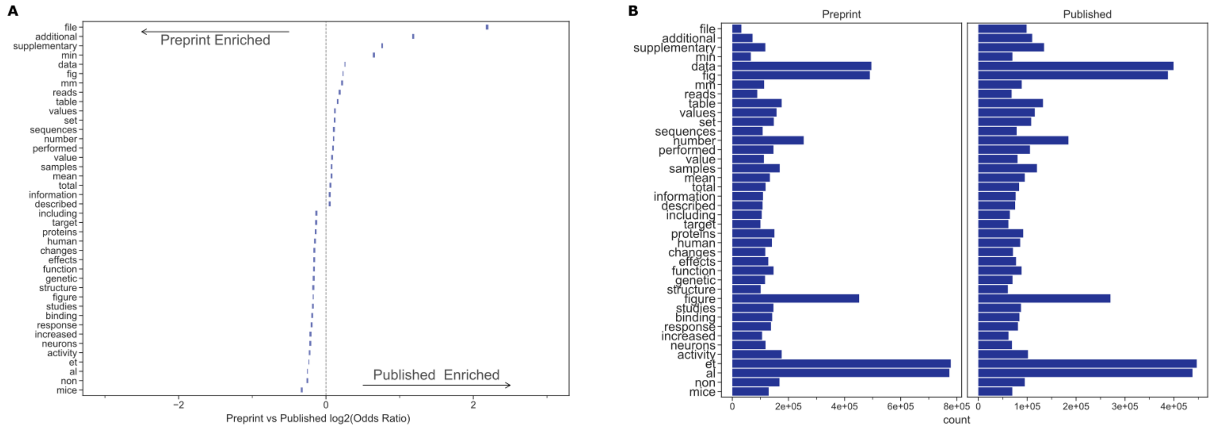

The word cloud of Figure 2B, C is somewhat nice as it shows the main words. However, this information could also be conveyed in the text. Would personally favor to quantitatively see loadings of first few principal components for different terms.

We have provided a table in our supplemental section (Supplemental Table 3 and 4). We also provide these tables below:

Supplementary Table S1: Top and bottom five cosine simularity scores between tokens and the PC1 axis.

**Cosine Simularity (PC1, word)**	**word**
0.6399154807185836	empirical
0.5995356000266072	estimates
0.5918321530159384	choice
0.5905550757923625	statistics
0.5832932491448216	performance
0.5803836474390357	accuracy
0.5757250459195589	weighting
0.5753027342288192	estimation
0.5730092178610916	uncertainty
0.5720493442813257	task
-0.4484093198386865	abrogated
-0.4490583645152233	transfected
-0.4500847285921068	incubating
-0.4531550791501111	inhibited
-0.4585422153514687	co-incubated
-0.4774721756292901	pre-incubated
-0.4793057689825842	overexpressing
-0.4839313193713342	purified
-0.4869885872803974	incubated
-0.5040798110023075	cultured

Supplementary Table S2: Top and bottom five cosine simularity scores between tokens and the PC2 axis.

**Cosine Simularity (PC2, word)**	**word**
0.65930201597598	genomic
0.6333515216782134	genome
0.5974018685580009	gene
0.5796531207938461	genomes
0.5353687686155728	annotation
0.5310140161149529	sequencing

0.5197350376908197	sequencesM.
0.5181781615670665	genome,
0.5168781637087506	bioinformatic
0.513853407439108	WGS
-0.4589201401582101	duration
-0.4690482252758019	stimuli
-0.4712875761979691	amplitudes
-0.4772723570301678	contralateral
-0.4813219679071856	stimulation:
-0.4946709932017581	delay
-0.5111990014804086	stimulus
-0.5251288188682695	amplitude
-0.543586881182879	stimulation
-0.5467022203294039	evoked

> The definition of "True matches" could be more explicit in within the main text as the preceding figure 3A could for some people set up a different anticipation.

We agree this term is too vague, and we have updated the text to be more explicit.

```
- Approximately 98% of our 200 pairs with an embedding distance in the
0-25th and 25th-50th percentile bins were scored as true matches (Figu
re 3B).

+ Approximately 98% of our 200 pairs with an embedding distance in the
0-25th and 25th-50th percentile bins were successfully matched with th
eir published counterpart (Figure 2B)
```

> The association given in Figure 4A seems to mainly stem from a few papers with large distances. Would an association be present when using the rank-based Spearman correlation instead of a linear regression? Would, for visualization, a logarithmic relationship describe the data better than a linear one?

This figure depicts the median half-life publication time for each preprint category within bioRxiv, so we don't think that it is driven by outliers within categories. We have adjusted the figure legend to more clearly note what we plotted.

> I believe that the analysis of Figure 4 B is quite clever as it would seem to address the thinkable concern of preprints with no delay and changes mainly stemming from those manuscripts that were already essentially accepted by manuscripts at the time of posting.

We are glad the reviewer found our analysis insightful.

> The analysis remarks that for the “Preprints in Motion Collection” the relationship between textual distance and time to publication disappears, and supports this through Figure 6E. However, the background trend in figure 6E includes publications that have been published at a time that exceeds a year. Hence a more faithful comparison would be to censor the background data by a distribution of durations that would correspond to the distribution of durations that would be possible for the “Preprint in Motions Collection” (taking distribution corresponding to interval between their dates on bioRxiv and the time at which authors assessed whether manuscripts were published).

This point is very interesting! The preprints in motion selection included preprints that were both posted within a specific time interval and then subsequently published. We think the best comparison set would be with preprints posted on the same dates as preprint in motion preprints, but where the analysis is conducted on the set after enough years have passed for those that will be published to have been published. Since we don’t have this set, we did conduct the analysis proposed by the reviewer. The results of this analysis are available in Supplemental Figure S6 (reproduced below). These results were qualitatively consistent with findings in the main text.

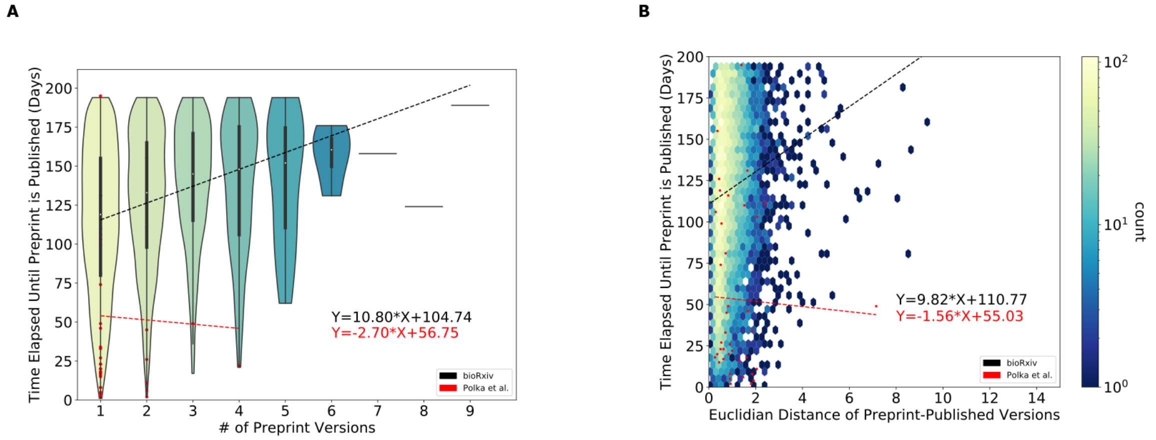

Adjusct Background Analysis

> Other:
>
> Labels within figures could often be increased in size to improve readability.

We have updated the size of our labels for this manuscript.

> The methods section briefly comments on some ambiguous cases for the matching. Would these cases be the result of modifications that defy a 1:1 mapping, e.g.: multiple stories getting fused, or one story getting split?

Out of our small set of disagreements, we encountered a variety of reasons for annotation mismatches. Some of these cases involved entirely different papers, which are clearly false positives. Other cases involved title, abstract or main text changes, while the remaining cases consisted of papers sharing similar research topics. We provide a table of these disagreements below along with short description about each pair.

biorxiv_doi_url	pmcid_url	Description
https://doi.org/10.1101/413450	https://www.ncbi.nlm.nih.gov/pmc/PMC2967545	Entirely different papers.
https://doi.org/10.1101/776930	https://www.ncbi.nlm.nih.gov/pmc/PMC6210049	Entirely different papers.
https://doi.org/10.1101/2020.01.13.905521	https://www.ncbi.nlm.nih.gov/pmc/PMC4171638	Text changes but same paper.
https://doi.org/10.1101/352963	https://www.ncbi.nlm.nih.gov/pmc/PMC6116183	Text changes but same paper.
https://doi.org/10.1101/513002	https://www.ncbi.nlm.nih.gov/pmc/PMC3545240	Similar aspects of research (liver studies) but different papers.
https://doi.org/10.1101/680843	https://www.ncbi.nlm.nih.gov/pmc/PMC6379322	Similar aspects of research (taxonomy studies) but different papers.
https://doi.org/10.1101/074450	https://www.ncbi.nlm.nih.gov/pmc/PMC5776756	Significant text changes but arguably same paper.
https://doi.org/10.1101/530758	https://www.ncbi.nlm.nih.gov/pmc/PMC6663035	Significant text changes

		but same paper.

> The results of Figure 2A could possibly be strengthened by avoiding Principal Components and replacing them by UMAP projects to account for non-linearity.

We chose to use PCA for figure 2A as our goal was to visually highlight the concepts captured by our generated principal components. We did also perform a UMAP embedding, which we included in this notebook but not the manuscript itself.

> Although peripheral to the current manuscript, their approach and data would also seem capable to providing an update-able map of the biomedical sciences, by applying their approach of Figure 2 to the PMC corpus data which the authors access too. Such a map could be interesting for those trying to obtain an overview about biology. In case that the authors do not hold plans to publish this elsewhere, and in case that it would be less than a day of work, I would recommend adding such a map to the supplement or as a web service.

Our web application provides 2D visualization of PMC's open-access corpus. This visualization uses SAUCIE, an autoencoder designed for RNA-seq, instead of UMAP or PCA to generate the landscape. We also constructed an auto-updater pipeline for this tool, incorporating new papers into our website and visualization every month. We think that the map is now in place in our server, and that others could produce their own map using either our API or the underlying SAUCIE models. Code to train our SAUCIE model can be found in this notebook and our fully trained model can be found using this link.

> Are the few publications in Figure 2A, which lie outside of the space that is generally occupied by their respective article categories, somewhat different when doing a superficial manual inspection (e.g.: misclassified by authors, or interdisciplinary research)

We sampled a select number of preprints from the neuroscience and bioinformatic category that were closer to the left side of the figure (PC1 <= -2.5 and -2 < PC2 < 2). We found that these outliers mainly consisted of interdisciplinary research (e.g., a bioinformatic paper analyzing fluorescence micrographs or a cell biology approach used to explore a neuroscience concept). We provide a table below of preprint DOIs that fall into this situation.

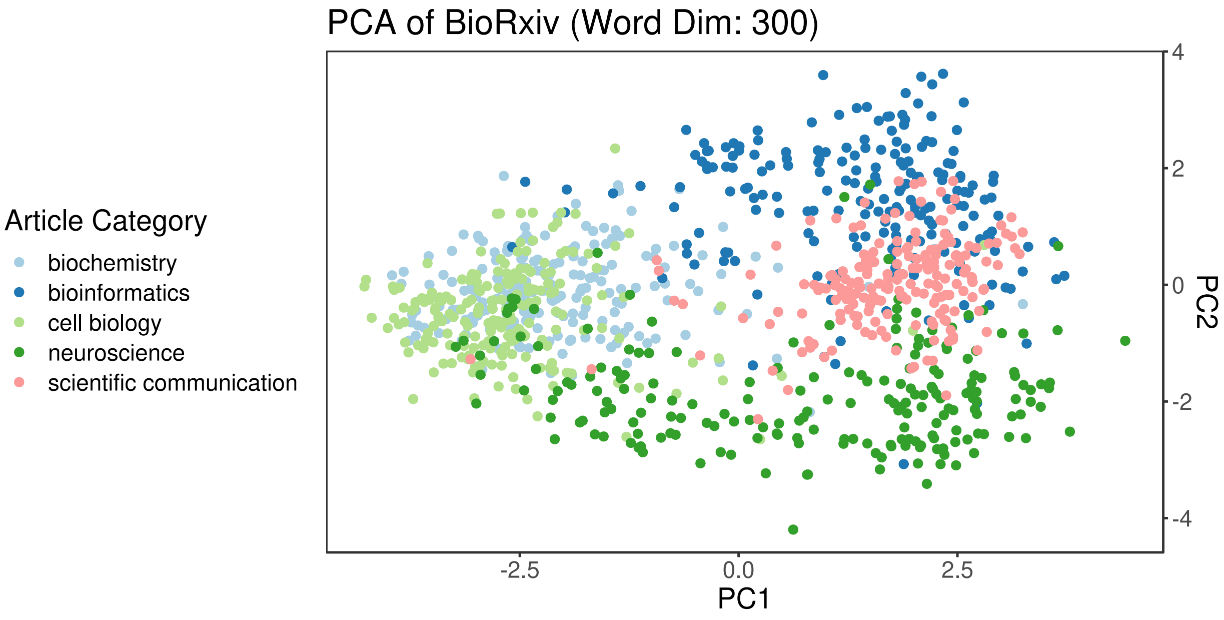

PCA plot

doi	document category
10.1101/075440	bioinformatics
10.1101/806216	bioinformatics
10.1101/696625	bioinformatics
10.1101/835181	bioinformatics
10.1101/583187	bioinformatics
10.1101/610196	neuroscience
10.1101/2020.01.08.898080	neuroscience
10.1101/664557	neuroscience
10.1101/655498	neuroscience
10.1101/244111	neuroscience

Adding a few words to “examining the top five and bottom five preprints” could avoid misunderstanding (e.g.: while I suspect that it is the position in Figure 2A, I was first thinking about the most/least successful/downloaded…)

We have updated the text to be more explicit.

```
- Examining the top five and bottom five preprints within the systems
biology field reinforces PC1's dichotomous theme (Table ...).

+ Examining the top five highest-scoring and bottom five lowest-scorin
g systems biology preprints along PC1 reinforces its dichotomous theme
(Supplementary Table ...).
```

> The vector representation of words and documents should allow the authors to quantify shifts that appear between preprints and published manuscripts. Though not necessary from my perspective, many interesting analyses could be done in vector space (e.g.: does language get more positive, or start to refer to more established concepts…?). Maybe there is something small that could be done. Alternatively, the discussion could possibly be extended to demonstrate the implications of vector space, and thus their own work, for future research into preprints and peer review.

Thank you for pointing out potential extensions to our vector space. We incorporated these suggestions into our discussion/conclusion section.

```
+ This embedding space could also be used to quantify sentiment trends
or other linguistic features.
+ Furthermore, methodologies for uncovering latent scientific knowledg
e [...] may be applicable in this embedding space.
```

> Along above, the discussion could be extended toward prior uses of Word2vec in the studies of science, such as Tshitoyan et al. Nature 2019.

We have added this citation into our discussion.

```
+ Furthermore, methodologies for uncovering latent scientific knowledg
e [...] may be applicable in this embedding space.
```

> Repeating the link to the web app in the main text would be convenient.

We have updated the text to include the web app link within our website section.

> Seeing Figure 6D and 6E, I would enjoy the authors showing or discussing more explicitly, whether textual differences increase with the number of revisions (and/or if there were some more complex changes such as reversions to earlier versions).

We agree with the reviewer that this would be interesting to examine. We performed a linear regression analysis to examine relationships between preprint version counts and the amount of change using all preprint-published pairs within bioRxiv. We found a small positive slope between version count and document distance (see below), but given the caveats involved with respect to small sample size at the extremes we elected not to include this analysis in the revised manuscript.

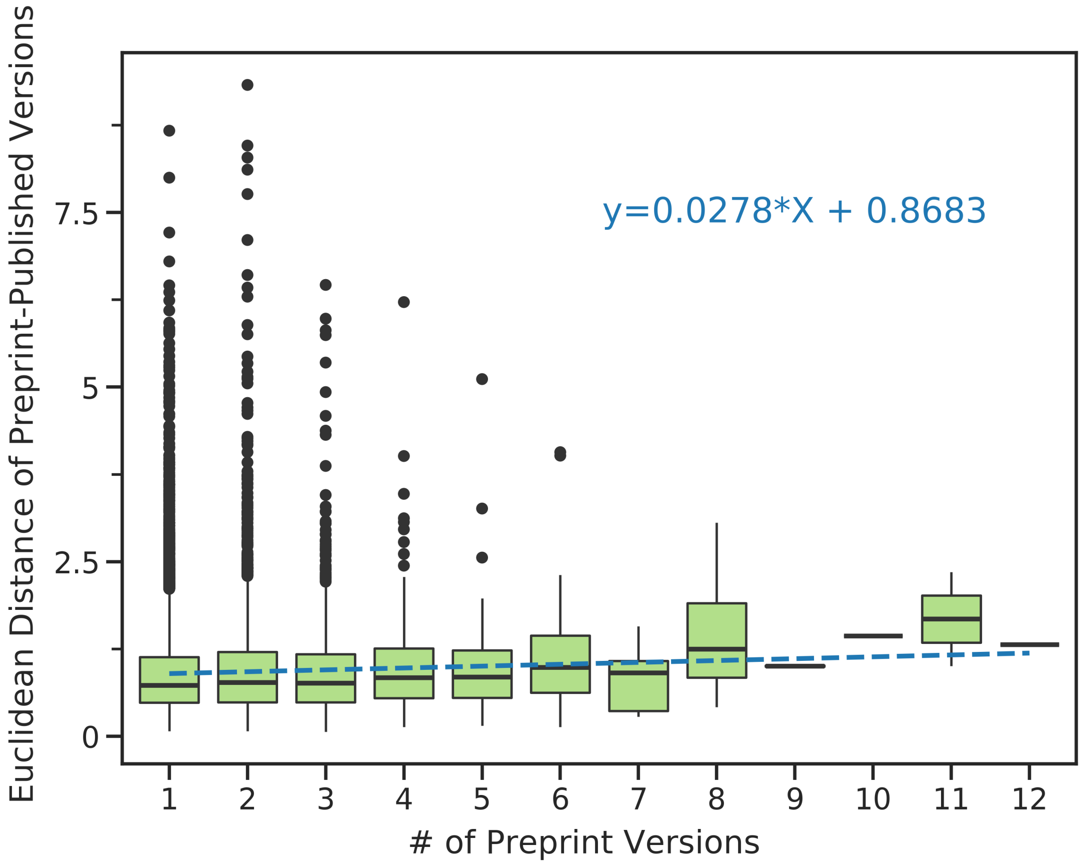

Version Count vs Document distance Linear regression

Reviewer #3

This study asks an important question: (how) do preprints change between their initial release on a preprint server and their eventual publication in a peer-reviewed journal? While the analysis of the linguistic changes doesn't reveal anything particularly exciting (mostly typesetting and references to supplementary information included in response to reviewer requests), this is an incredibly useful result in demonstrating that preprints are typically of high quality, which has broad implications for how researchers and their work are assessed in career, funding, and publishing decisions. The authors have developed some very promising deliverables based on document embeddings that should be broadly applicable to readers, authors, journal editors, and other stakeholders navigating the complex landscape of preprinted and published literature.

We appreciate the reviewer's positive comments on the value of our manuscript. We agree that the linguistic changes aren't particularly exciting and with the reviewer's sentiment that that finding, in itself, is exciting.

Major Comments:

The method for discovering unannotated preprint-publication relationships is very neat, but I imagine it's rather unwieldy to match a novel publication against the full-text bioRxiv corpus in downstream applications (e.g., bioRxiv's automation)–could this be optimized by reducing the search space to preprints that share some or all of the same authors, within a reasonable date range, and/or only considering paper/preprint metadata (e.g., abstract, title, references)? Such an approach might also enable annotation of preprints that are eventually published as non-OA peer reviewed articles for which such metadata are available.

We investigated this based on the reviewer's question. Our results suggest that it is likely to be feasible to identify preprint-published pairs using abstracts alone. We generated document embeddings using solely abstracts and calculated distances between known preprint-published pairs and preprints with a randomly sampled article from the same journal. We found that the ranking by abstract distances was slightly better than full text (figure below) for matching preprints with their published pair. This indicates that abstracts can be used to establish preprint and published links, and while the evidence is relatively weak (as the difference between full text and abstract is small) it suggests that perhaps abstracts undergo less change than full text.

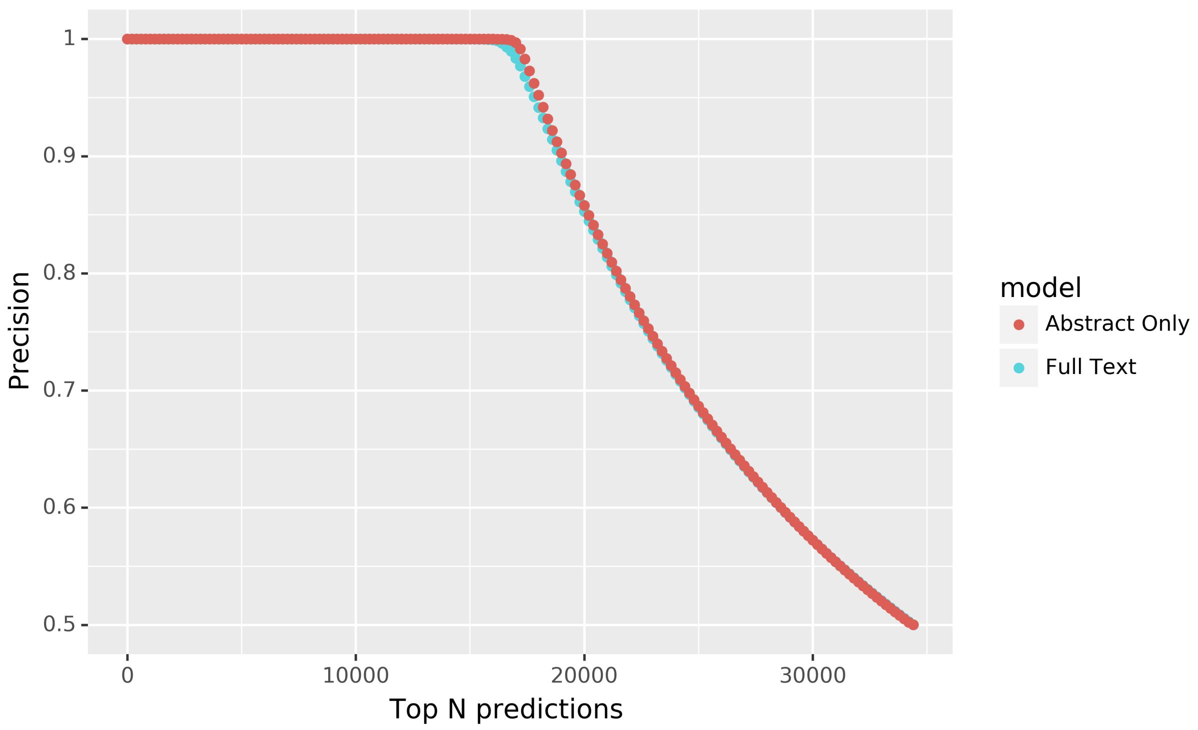

Abstracts vs Full Text

Section "Building Classifiers to Detect Linguistically Similar Journal Venues and Published Articles":

"Specific journals publish articles in a focused topic area, while others publish articles that cover many topics. Likewise, some journals have a publication rate of at most

> hundreds of papers per year, while others publish at a rate of at least ten thousand papers per year. Accounting for these characteristics, we designed two approaches - one centered on manuscripts and another centered on journals.” << this could use some unpacking and/or reorganizing of details found later in this section–as I understand it, the variation in journals’ topical breadth motivates the development of a manuscript-focused classifier (so that topically similar papers appearing in generalist journals do not get obscured) and the variation in journals’ publication rates motivates a journal-focused classifier (so that high-output journals do not overwhelm more selective or less popular journals).

We agree with the reviewer that this section was unduly dense, and we have revised the manuscript to more clearly unpack this explanation.

```
+ Training models to identify which journal publishes similar articles
is challenging as not all journals are the same.
+ Some journals have a publication rate of at most hundreds of papers
per year, while others publish at a rate of at least ten thousand pape
rs per year.
+ Furthermore, some journals focus on publishing articles within a con
centrated topic area, while others cover many dispersive topics.
+ Therefore, we designed two approaches to account for these character
istics.
+ Our first approach focuses on articles that account for a journal's
variation of publication topics.
+ This approach allows for topically similar papers to be retrieved in
dependently of their respective journal.
+ Our second approach is centered on journals to account for varying p
ublication rates.
+ This approach allows more selective or less popular journals to have
equal representation to their high publishing counterparts.
```

> I’m also curious how often these two classifiers agree–are the top matching papers typically found in the top matching journals? In cases where the two classifiers tend to disagree, are there any common characteristics of the preprints the application is trying to classify?

We evaluated our classifier agreement by calculating the overlap coefficient, which is designed to measure the overlap between two sets. We randomly sampled 1700 out of 20232 known preprint-published pairs from our test dataset. We generated ten recommendations from our centroid classifier and ten unique journal recommendations from our paper-paper model for every sampled pair (as the paper classifier can return papers from the same journal, this means we are examining ten or more manuscripts until we reach ten unique journals). This resulted in an average overlap coefficient of 0.21. Along with this calculation, we generated baselines for each model. Our first baseline was designed for our journal centroid model. We randomly sampled ten journals without replacement for each preprint-published pair. We compared this random listing against our original journal centroid recommendation list and found an average score of

0.0184. Our other baseline was designed for our paper-paper classifier. We randomly sampled without filtering ten unique journals for each preprint-published pair and compared this sample to the original paper-paper recommendation list. This baseline overlap coefficient was 0.009. Our takeaway from this analysis is that both approaches agree much more than they would due to random overlap, but the overlap coefficient remains modest. Because of the relatively large number of discrepancies between the resulting sets, we were not able to identify a practical way to answer the characteristics of preprints that led to differences vs common predictions.

Minor Comments (by section):

Introduction:

The references of text mining on biomedical corpora should include Desai et al (2018) [https://www.biorxiv.org/content/10.1101/333922v1.abstract], which describes a similar recommendation engine.

We agree and have added this reference.

```
- Textual analysis uses linguistic, statistical, and machine learning
techniques to analyze and extract information from text [...].

+ Textual analysis uses linguistic, statistical, and machine learning
techniques to analyze and extract information from text [...].
```

Section “Comparing Corpora”:

Inconsistent formatting of “spaCy”

We updated our manuscript to make sure spaCy is formatted correctly.

```
- Spacy is a lightweight and easy-to-use python package designed to pr
eprocess and filter text [@spacy2].

+ SpaCy is a lightweight and easy-to-use python package designed to pr
eprocess and filter text [@spacy2].
```

Define “stopwords,” since many readers may be unfamiliar with this term

We added an explanation of “stopwords” to the manuscript.

```
+ All corpora contain multiple words that do not have any meaning (e.g
. conjunctions, prepositions, etc.) or occur with a high frequency.
+ These words are termed stopwords and are often removed to improve te
xt processing pipelines.
```

Section “Constructing a Document Representation for Life Sciences Text”:

This switches back to using “words” instead of “tokens” as in the previous section

Thank you for pointing out this inconsistency. We have carefully edited our manuscript to make sure “tokens” was consistently used instead of “words”.

Section “Measuring Time Duration for Preprint Publication Process”:

Does this include the new preprint-publication pairs discovered in the previous section, or only those annotated in the data provided by the bioRxiv API?

This section only contains preprints pairs that have been established before our document matching approach.

Section “Preprints with more versions or more text changes took longer to publish”:

Fig. 4: can the longer publication times for scicomm/education papers (Fig 4a) be explained by a tendency to go through more versions (Fig 4b)?

We investigated this. Unfortunately, we were not able to provide a detailed answer to this question as most published articles in this category weren’t contained in Pubmed Central’s Open Access Corpus (PMCOA). We do provide a table of preprints that have a matching counterpart in PMCOA in our supplemental files, which provides the ingredients for further investigations.

It might be worthwhile to explore what happens post-publication to papers that go through more preprint revisions and take longer to publish, as this could have practical implications for authors as they decide when/if to submit/revise their preprints. Do these papers ultimately receive more citations, end up in journals with higher impact factors, or receive more attention on social media?

We agree that this is an interesting question. We thought that this work would be outside the scope of this manuscript, but we wanted to make it as easy as possible for this to be tackled in the future. We provide a supplemental file (published_preprints_information.xlsx) containing preprints and corresponding publication to enable these future studies.

Section “Preprints with similar document embeddings share publication venues”:

From personal experience, converting bioRxiv PDFs to text sometimes introduces weird noise and artifacts. Since bioRxiv and medRxiv both offer full-text HTML for many (if not all?) articles, is it possible to modify the application to use this cleaner data source?

At the time of submission, we recognized that using XML was better than solely relying on a pdf parser. We have now updated the webserver to attempt to retrieve the XML version first, then resort to the pdf parser if the XML version is unavailable.

Section “Contextualizing the Preprints in Motion Collection”:

Figure description for Fig 6E is mislabeled as D

We updated our text to fix this label mismatch.

There are several casually/awkwardly-worded or grammatically incorrect sentences throughout that could use some finesse:

Introduction:

“We hypothesize that preprints and biomedical text are pretty similar…”

Measuring Time Duration for Preprint Publication Process:

“Preprints that are published can take varying amounts of time to be published.”

“We accomplish this by first randomly sampled with replacement a pair of preprints…”

Building Classifiers to Detect Linguistically Similar Journal Venues and Published Articles:

“Preprints are more likely to be published in journals that contained similar content to work in question.”

Web Application for Discovering Similar Preprints and Journals:

“The application downloads a pdf version of any preprint hosted on the bioRxiv or medRxiv server uses PyMuPDF to extract text from the downloaded pdf and feeds the extracted text into our CBOW model to construct a document embedding representation.”

Preprints with more versions or more text changes took longer to publish:

“Each new version adds additional 51 days before a preprint is published.”

We have updated these sentences in the manuscript. We thank the reviewer for providing these corrections.

```
- We hypothesize that preprints and biomedical text are pretty similar
, especially when controlling for the differential uptake of preprints
across fields.

+ We hypothesize that preprints and biomedical text will appear to hav
e similar characteristics, especially when controlling for the differe
ntial uptake of preprints across fields.

- Preprints that are published can take varying amounts of time to be
published.
```

+ Preprints can take varying amounts of time to be published.

- We accomplish this by first randomly sampled with replacement a pair of preprints from the Bioinformatics topic area as this was well represented within bioRxiv and contains a diverse set of research articles.

+ We first randomly sampled with replacement a pair of preprints from the Bioinformatics topic area as this was well represented within bioRxiv and contains a diverse set of research articles.

- Preprints are more likely to be published in journals that contained similar content to work in question.

+ Preprints are more likely to be published in journals that publish articles with similar content.

- The application downloads a pdf version of any preprint hosted on the bioRxiv or medRxiv server uses PyMuPDF [...] to extract text from the downloaded pdf and feeds the extracted text into our CBOW model to construct a document embedding representation.

+ Our application attempts to download the full text xml version of any preprint hosted on the bioRxiv or medRxiv server and uses the lxml package (version num) to extract text.
+ If the xml version isn't available our application defaults to downloading the pdf version and uses PyMuPDF [...] to extract text from the pdf.
+ The extracted text is fed into our CBOW model to construct a document embedding representation.

- Each new version adds additional 51 days before a preprint is published.

+ Every additional preprint version was associated with an increase of 51 days before a preprint was published.
